# An Essential Role of Gelatin in the Formation Process of Curling in Long Historical Photos

**DOI:** 10.3390/polym13223894

**Published:** 2021-11-11

**Authors:** Jiaojiao Liu, Yuhu Li, Daodao Hu, Xiaolian Chao, Yajun Zhou, Juanli Wang

**Affiliations:** Engineering Research Center of Historical Cultural Heritage Conservation, Ministry of Education, School of Materials Science and Engineering, Shaanxi Normal University, Xi’an 710119, China; liujiaojiao@snnu.edu.cn (J.L.); daodaohu@snnu.edu.cn (D.H.); chaoxl@snnu.edu.cn (X.C.); zhouyajun@snnu.edu.cn (Y.Z.)

**Keywords:** long historical photos, curling, gelatin, paper base layer, preservation

## Abstract

Curling disease in long historical photos significantly affects the presentation of cultural heritage information. However, people lack attention to the formation process and microstructural changes of photo curling. In this article, a long historical photo (1912–1949 AD) collected by the Second Historical Archives of China was taken as the research object, and the formation process and cause of the curling were further explored. Firstly, Fourier-transform infrared spectroscopy (FTIR), X-ray diffraction (XRD), scanning electron microscope (SEM), X-ray energy disperse spectrometer (EDS), and other instruments were used to analyze the material composition of the long historical photo. It was found that the photographic paper was made of gelatin, barium sulfate, and plant fiber layers. Then, the effects of hygrothermal environments on curling and contraction in the gelatin layer and simulated photographic paper were explored. Meanwhile, the formation process and main influence factors of the curling were preliminarily revealed. The morphological analysis by SEM was carried out to identify the inner correlation between the microstructure and curling of photos. Finally, the possible formation cause of photo curling was analyzed. This study provides a scientific basis and experimental data for the preservation and restoration of long historical photos based on gelatin.

## 1. Introduction

**Figure 1:**
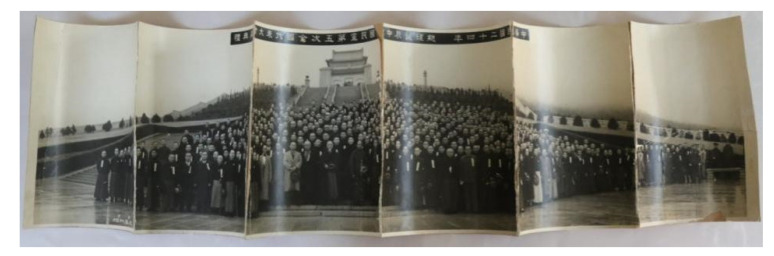
Typical damage of curling disease in a group photo of Fifth National Convention of the National Army in the 24th year of the China.

Photos are important photosensitive image archives that directly witness and corroborate major historical events, historical figures, and social reforms. They are extremely precious modern cultural heritages and world memories. Since 1880, gelatin has been extensively used for the preparation of photographic materials thanks, e.g., to good dispersing properties. Among them, the silver salt gelatin process is the most common. However, gelatin film is highly susceptible to diseases, such as damage [1], cracking and crazing [2,3,4], watermarks and mildew spots [5,6], fading [7,8], and scratches [9]. Brittle curling is a special disease that is frequently found in long protein silver salt photos, which seriously affects the preservation and information safety of photos (Figure 1).

The influence of environmental temperature and humidity on the stability of coated paper has attracted extensive attention in recent years [10,11,12,13,14,15]. A hydrophobic coating on paper improves its water tolerance and printing performance, which causes the coated paper to curl easily in alternate wetting–drying environments [16,17,18]. Curling can be inhibited by maintaining stable water content through paper isolation, which can be achieved by applying a waterproof layer on the non-printed surface or sizing on the non-coated surface. For repairing and preservation of paper-based photos, the Smithsonian Institution (Washington, DC, USA) suggested maintaining low-temperature and low-humidity conditions on a long-term basis [19]. On the other hand, digital storage offers an effective way of permanently preserving the image information, and this approach has been internationally recognized [20,21,22,23,24]. So far, digital technology is used to repair photos with slight curling, deformation, or creasing, thus greatly improving the image quality. Our team has been involved in preserving black-and-white historical photos for many years and has achieved some important results in terms of scratches, mildew, fading, toughening, and flattening [25,26,27,28,29]. These important studies have offered fundamental inspiration and laid a solid foundation for the prevention and preservation of historical photos. However, brittle curling is widespread in long historical photos and poses serious threats to the information safety of cultural relics. As far as we know, there has been no previous research on the cause analysis of curling in long historical photos, and the literature on repair methods is also very limited. Therefore, research on the formation process and cause of curling in historical photos is particularly important for the effective elimination of curling disease and better preservation of long historical photos.

This study is aimed at exploring the cause of curling in long historical photos and providing a scientific basis for better preservation of gelatin paper-based photos. Some gelatin paper-based historical photos from the China (1912–1949) were collected by the Second Historical Archives of China, including photos with varying degrees of curling, creasing, and fracture. Some of them were used for the first time to investigate the formation process and cause of curling. SEM, EDS, FTIR, and XRD were employed to analyze the microstructure and material composition of the long historical photo. Simulated photographic paper samples with varying degrees of curling and contraction were prepared by altering hygrothermal environments, and the effects of hygrothermal environments on regular curling changes were also studied. The inner correlation between the microstructures of photos and the macroscopic changes in curling was revealed by freeze-drying the simulated samples and observing their surface morphologies by SEM. This study provides theoretical support for identifying the formation cause of curling in long historical photos.

## 2. Experimental Section

### 2.1. Characterization of the Structural Composition of Photos

The micromorphology and elemental composition of the photographic paper interface was investigated by SEM and EDS. The characteristic functional groups and molecular diffraction peaks of molecular functional groups in each layer were analyzed with FTIR and XRD. Tested details were as follows:

(1) SEM-EDS: A sample (5 × 5 mm^2^) that contained no information was chosen at the edge of the photo relics and placed on double-sided sticky tape on an aluminum SEM specimen holder. The surface of the sample was sprayed with gold 80s by an ion sputtering apparatus (SCD005, Baltek, Liechtenstein, Germany). The sample was examined using SEM (Quanta 200, FEI, Columbus, OH, USA). Analyses were performed at a high vacuum with an accelerating voltage of 20 kV. Elemental spectrums were generated with a Quanta 200 SEM. Magnification was 4000×.
(2) FT-IR spectroscopy: Fourier transform infrared (FT-IR) coupled with a diamond ATR method was used in the reflection mode to analyze the composition of the samples at room temperature and ambient humidity. FT-IR (Vertex 70, Bruker, Karlsruhe, Germany) analysis of each layer was conducted using PerkinElmer Spectrum Two in the range between 4000 and 500 cm^−1^ with a resolution of 4 cm^−1^, and the scan number was 40 times. In order to reduce the effect of carbon dioxide and water vapor on FT-IR spectra, the sample spectra have removed the background contributions.
(3) XRD: The sample was a small piece of the surface of a paper base after the gelatin emulsion layer had fallen off (about 5 × 5 mm^2^). XRD (Smart Lab, Rigaku Corporation, Japan) was used to test the crystalline structure of the sample from the photo relics. Test condition: The X-ray intensity was 45 kV/200 mA, scanning speed was 5°/min, and scanning range was 15–40°.

### 2.2. Preparation of Simulated Photographic Paper Samples

Photographic gelatin was prepared into an 8% aqueous solution, and the gelatin solution was evenly coated on the barium photographic paper surface with a quantitative coating stick. The gelatin solution was dried naturally at room temperature. Then, it was cut into several strips of 12 cm × 2 cm size. The thin and uniform gelatin film was prepared with a polytetrafluoryl grinding tool and dried naturally. The paper base and gelatin film samples were subjected to dry and wet cycles at room temperature and high temperature several times, respectively. To obtain the simulated samples with different contraction rates and curling degrees, we set the normal temperature and high temperature at 25 °C and 45 °C, respectively; the dry and wet humidity at 18% and 75%, respectively; and the dry and wet time at 24 h. Regular changes in the simulated samples with curling and contraction were then used to study the effects of ambient damp-heat.

### 2.3. Micromorphological Characterization of Gelatin Films and Paper Base Layers

The simulated samples of gelatin films with different shrinkage and paper bases with different curling were soaked in distilled water for 5 min, placed in the freeze-dryer for 48 h, then observed under SEM to visualize the morphological changes.

## 3. Results and Discussion

### 3.1. Structures and Composition of Long Historical Photo

**Figure 2:**
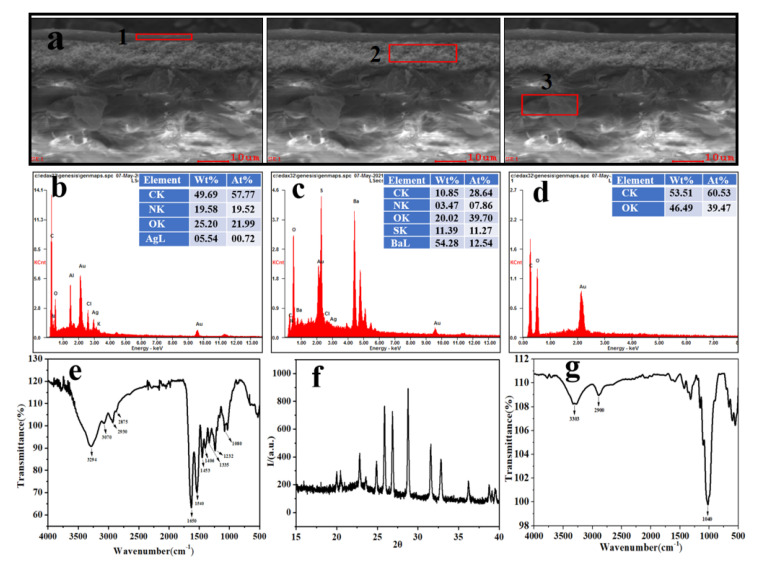
Material composition analysis of each layer in the long historical photo: SEM cross-section of each layer (1–3) (**a**), corresponding energy spectra (**b**–**d**), and infrared spectra (**e**–**g**).

As shown in Figure 2, morphological analysis by SEM indicates the microstructural characteristics of the historical photo from the China. SEM images reveal that the historical photo has an obvious layered structure. The composition of each layer was analyzed by EDS, and results show that layer 1 is composed of C, N, O, Ag, and Cl. FTIR analysis of layer 1 indicates that a broad peak at 3600–3000 cm^−1^ is attributed to the OH bond. In addition, amide A (3330–3310 cm^−1^) and amide B (3070 cm^−1^) bands also appear in the same region [30,31]. The amide I band contains stretching vibrations of carbonyl groups at 1650 cm^−1^, whereas the amide II band contains a CN stretching vibration and in-plane NH distortion absorption vibration peak at 1550 cm^−1^. The amide III bands show carbon–nitrogen stretching vibrations at 1280 cm^−1^ [32]. Comprehensive EDS-IR analysis revealed that the main surface ingredients of layer 1 are gelatin and silver chloride photosensitive materials.

EDS analysis of layer 2 shows that the main elements of this layer include C, N, O, Ba, and S. Correspondingly, there are intense and sharp diffraction peaks, indicating a higher degree of crystallinity. The particulate matter matches with JCPDS card (i) 24–1035, and its powder X-ray diffraction pattern is matched with the BaSO_4_ orthorhombic system. Comprehensive EDS-XRD analysis reveals that layer 2 is mainly a mixture of BaSO_4_ and gelatin. EDS analysis of layer c reveals C and O as the main elements of this layer. Three FTIR peaks at 2900 cm^−1^, 3400 cm^−1^, and 1040 cm^−1^ are typical characteristic peaks of paper cellulose. The above results show that this historical photographic paper has two characteristics. Firstly, in terms of composition, it has an obvious layered structure. Secondly, gelatin is the primary film-forming matter of the emulsion and baryta layers. We inferred that these characteristics and structural differences would cause curling of photos in alternate wetting–drying environments. The above results suggest that the long historical photo with brittle curling from the China is mainly made of a gelatin silver salt paper base. This result offers a research basis for subsequent cause analysis of curling.

### 3.2. Macroscopic Effects of Damp-Heat Factors on Curling and Contraction in Simulated Photographic Paper Samples

#### 3.2.1. Effects of Damp-Heat Factors on Curling Changes in Simulated Photographic Paper Samples

**Figure 3:**
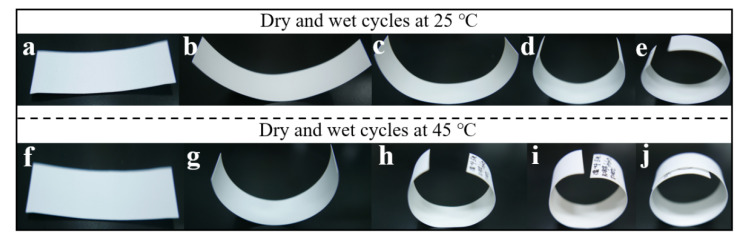
Pictorial diagram of regular curling changes in the simulated photographic paper samples prepared under dry (relative humidity: 18%)–wet (relative humidity: 75%) cycles: untreated (**a**,**f**), one dry–wet cycle (**b**,**g**), three dry–wet cycles (**c**,**h**), five dry–wet cycles (**d**,**i**), and seven dry–wet cycles (**e**,**j**).

Paper base (porous fiber polymer) and gelatin layers (compact hard film) are both hygroscopic materials. Gelatin is usually characterized by high hygroscopicity and expansibility. In this study, we speculated that the differences in damp-heat properties of the paper base and gelatin caused serious curling. Therefore, regular changes in the curling degree of the simulated photographic paper sample were studied by controlling the numbers and parameters of high-temperature dry–wet cycles (Figure 3). The glass transition temperature (T_g_) of gelatin drops to room temperature (23 °C) when the gelatin has equilibrated to a relative humidity condition of approximately 75% [19]. Gelatin shows one conversion from a triple-helical structure to a random coil configuration when the gelatin is subjected to high relative humidity and temperature (>T_g_) conditions [33]. According to the site survey, the temperature and humidity upper limit in the storeroom for the collection of historical photos were about 45 °C and 75%, respectively. To study the effects of ambient damp-heat on curling changes in simulated photographic paper samples, we chose the temperatures of 25 °C and 45 °C (>T_g_) and high relative humidity of 75%. Simulated photographic paper samples were put through normal-temperature (25 °C) and high-temperature (45 °C) dry–wet cycles one, three, five, and seven times. The photographic paper curled to varying degrees towards gelatin layers under different dry–wet cycles and high-temperature dry–wet cycles accelerated the curling. These tests provide scientific facts for the study of the photo curling formation mechanism. The curling phenomena further suggest that the formation of photo curling is closely related to damp-heat environments and that gelatin plays a dominant role in the curling formation.

#### 3.2.2. Effects of Damp-Heat Factors on Contraction Rates of Paper Base Layers and Self-Supported Gelatin Films

**Figure 4:**
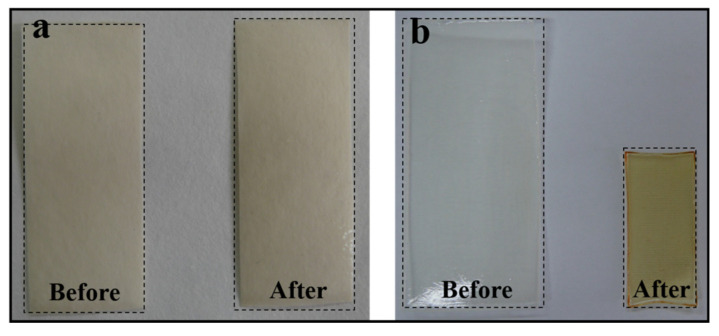
Contraction changes in paper base (**a**) and gelatin films (**b**) under accelerated alternate damp-heat environments. Before: untreated, After: seven dry (RH = 18%) and wet (RH = 75%) cycles at 45 °C.

To further validate the above speculation, we put the paper base and gelatin films under alternate high-temperature dry–wet environments with 14 cycles (Figure 4). Results indicate that the paper base is relatively stable and only incurred slight contraction changes, whereas self-supported gelatin films incurred dramatic contraction changes in damp-heat environments. These results are consistent with the above speculation.

#### 3.2.3. Effects of Damp-Heat Cycles on the Contraction Rate of Self-Supported Gelatin Films

**Figure 5:**
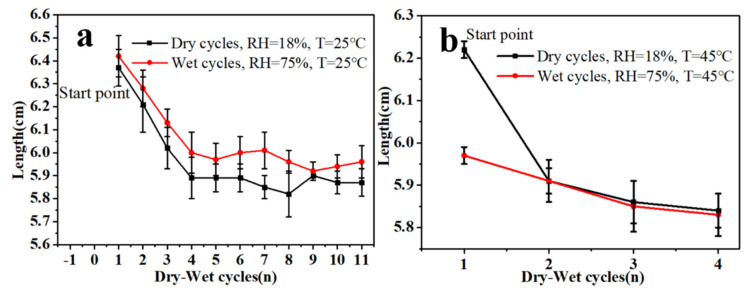
Contraction changes in self-supported gelatin films under dry–wet cycles at 25 °C (**a**) and 45 °C (**b**). RH: relative humidity.

Photos are sometimes subjected to alternate damp-heat and dry-heat changes during preservation. To explore the specific contraction trends of self-supported gelatin films in damp-heat and dry-heat environments, we placed gelatin films under normal-temperature and high-temperature environments with dry–wet cycles. The gelatin films (all in initial natural drying state) were kept in a normal-temperature high-humidity environment to observe their contraction changes. As shown in Figure 5a, the gelatin films showed continuous hygroscopic expansions and dry contraction changes and finally remained in a constant contraction state after multiple cycles.

In high-temperature dry–wet cycles, gelatin films exhibited obvious contraction changes during the first cycle from the initial dry environment to a high-temperature high-humidity environment (Figure 5b). Then, they exhibited a constant contraction state in subsequent cycles. These results suggest that high temperature intensifies the contraction changes in gelatin films during dry–wet cycles, directly validating the aforementioned speculation. The contraction changes of gelatin films in high-humidity environments constitute the primary cause of serious brittle curling in photos.

### 3.3. Effects of Damp-Heat Factors on the Micromorphology of Curled Samples

**Figure 6:**
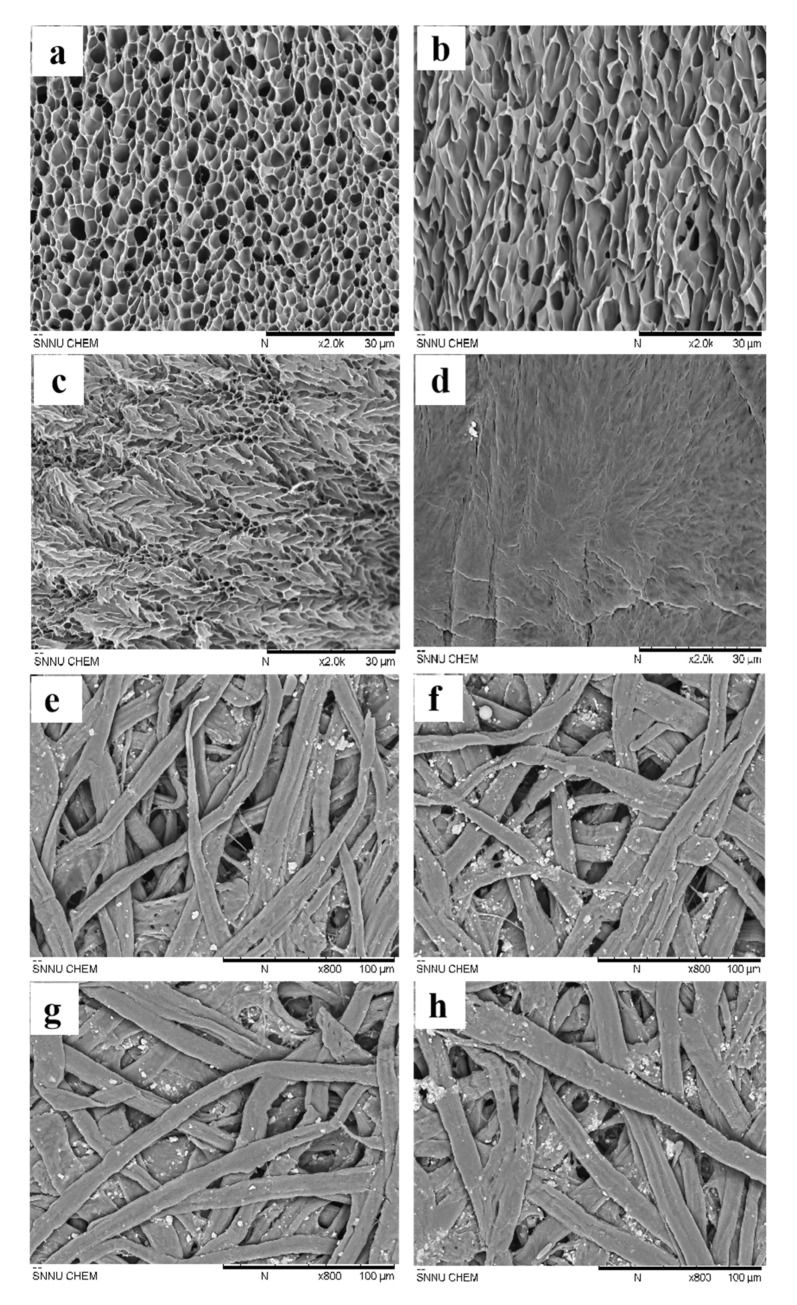
Surface morphology of the gelatin films and (**a**–**d**) and the paper base (**e**–**h**) with different micro-porous structures: untreated (**a**,**e**), one damp-heat cycle (**b**,**f**), three damp-heat cycles (**c**,**g**), and seven damp-heat cycles (**d**,**h**).

To study the microstructures of the gelatin films and paper base layers treated under different high-temperature dry–wet cycles, they were freeze-dried after hygroscopic expansion, and their SEM test results are shown in Figure 6. As shown in Figure 6a, the untreated gelatin film exhibited an obvious porous structure after freeze-drying. In Figure 6b–d, the porous structure gradually narrowed after damp-heat cycles, suggesting that the macroscopic contraction changes of gelatin films are closely related to the aggregation of molecular skeletons in gelatin polymer. Figure 6e–h show that the paper base fiber samples did not display an obvious change in the surface structure after freeze-drying. These results indicate a significant difference between the gelatin and the paper base layer in their contraction behaviors under alternate damp-heat environments. Compared to the paper base layer, the gelatin layer displayed more abrupt contraction changes, thereby causing the photos to curl towards the gelatin layers.

### 3.4. Formation Cause of Photo Curling

**Figure 7:**
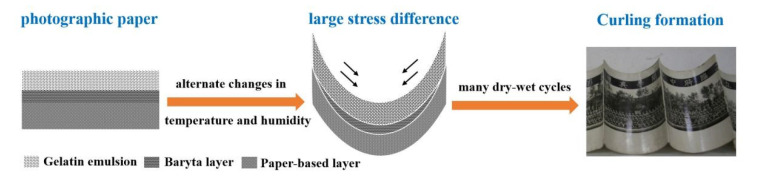
The possible formation process of historical photo curling.

According to the above research results, it is found that photo curling is related to the aging of emulsion gelatin under damp-heat cycles and that alternate damp-heat changes are the most significant factor affecting the curling and fracture in gelatin photos. The possible curing mechanism in historical photos is illustrated in Figure 7. The formation of curling in historical photos can be explained from the following aspects. Firstly, from the structural composition of the photographic paper from the China, gelatin is the primary film-forming material in the gelatin protection layer, emulsion layer, and baryta layer. Gelatin films possess high hygroscopicity, low thermal stability, and poor mechanical property (brittleness) [34,35]. Secondly, during hygroscopic expansion and dry contraction, the aggregation behavior of gelatin supramolecules produces different contraction stress in each layer. The stress in the gelatin layer is greater than that of the paper base layer, causing the curling towards the gelatin layer. Thirdly, in case of alternate changes in temperature and humidity levels, especially in high-temperature dry–wet environments, there is a large stress difference between gelatin films and paper base layers, leading to serious brittle curling or fracture.

## 4. Conclusions

In this study, the formation cause of curling in the group photo of the Fifth National Convention of the National Army in the 24th year of the China (1935), collected by the Second Historical Archives of China, was analyzed. According to SEM, EDS, XRD, and FT-IR results, photographic paper material has an obvious layered structure, and gelatin is the primary film-forming matter in their photosensitive emulsion and baryta layers. The results of damp-heat tests on photographic paper, paper base, and gelatin films suggest that the formation of photo curling is closely related to damp-heat environments, and that gelatin plays a dominant role in curling formation. The contraction changes in gelatin under high-temperature dry-wet environments are the primary causes of serious brittle curling in photos. According to the morphological analysis by SEM, the aggregation of gelatin supramolecules induced by damp-heat environments caused a collapse in their porous structure at the microscopic level, thereby leading to macroscopic contraction and photo curling. These research results are consistent with the formation process of brittle curling in photos. This work presents that alternation between high-temperature and wetting–drying is a critical factor affecting the degradation rate of gelatin, hence improving storage environments constitutes the most important link in the preservation of gelatin paper-based long historical photos.

## Data Availability

The data presented in this study are available on request from the corresponding author.
